# Association between depression and heart failure: A meta-analysis of cohort studies with 2.6 million participants

**DOI:** 10.12669/pjms.41.10.12723

**Published:** 2025-10

**Authors:** Haiying Gu, Lu Zhu, Qiuxia Wang

**Affiliations:** 1 Haiying Gu, Department of Psychiatry, Huzhou Third Municipal Hospital, the Affiliated Hospital of Huzhou University, Huzhou, Zhejiang Province 313000, P.R. China; 2 Lu Zhu, Department of Psychiatry, Huzhou Third Municipal Hospital, the Affiliated Hospital of Huzhou University, Huzhou, Zhejiang Province 313000, P.R. China; 3 Qiuxia Wang, Department of Psychiatry, Huzhou Third Municipal Hospital, the Affiliated Hospital of Huzhou University, Huzhou, Zhejiang Province 313000, P.R. China

**Keywords:** Cardiac failure, Cohort, Depression, Mental disorders, Psychiatric disorders

## Abstract

**Objective:**

Depression is a widely prevalent mental disorder that has been linked to several systemic diseases. While a relationship has been established between depression and cardiovascular diseases, the risk of heart failure (HF) in depressed patients is unclear. Herein, we collated evidence from cohort studies to establish the link between depression and HF.

**Methodology:**

PubMed, Embase, CENTRAL, Web of Science and Scopus were searched up to 28 July 2024 for cohort studies excluding baseline HF patients and reporting adjusted effect size of the association between depression and HF after a minimum follow-up of five years. A random-effect meta-analysis was conducted.

**Results:**

Eleven studies with 2,635,205 participants were included. Pooled analysis showed that depression was a statistically significant risk factor for the development of HF (HR: 1.25 95% CI: 1.13, 1.38 I^2^=87%). Results did not alter in significance on sensitivity analysis. There was minimal change in the significance of results on subgroup analyses stratified by location, design, included participants, method of assessment of depression and HF, prevalence of depression, incidence of HF and follow-up duration. Meta-regression analysis based on moderators diabetes mellitus, hypertension, current smoking and mean body mass index did not reveal significant results. GRADE assessment indicated that certainty of evidence was “very low”.

**Conclusions:**

Very low quality of evidence suggests that there may be an association between depression and development of HF. Appropriate screening should be undertaken for early recognition of HF in patients with depression.

***Registration No:*** (PROSPERO CRD42024573423).

## INTRODUCTION

Heart failure (HF) is a common cardiovascular disease (CVD) characterized by a structural and/or functional cardiac aberration mostly seen with heightened natriuretic peptide levels with or without objective signs of pulmonary or systemic congestion.^1^ The prevalence of HF has reached epidemic proportions with about 64.3 million adults affected by the disease around the globe. Data indicates that around 1-2% of the Western population is diagnosed with HF.^2^

HF has a varied etiology and can be caused by genetic defects, metabolic disorders as well as systemic diseases but atherosclerotic heart disease and hypertension (HT) are among the most frequent causes.^3^ Given the heightened prevalence of lifestyle-related illnesses globally in the modern era, there seems to be an increasing trend of HF in the coming decades.^4^ While there have been advancements in imaging modalities and the emergence of several pharmacological and non-pharmacological interventions for HF,^5^ survival remains poor and about one in three patients does not survive within the first year of diagnosis.^6^ Long-term mortality rates (>1 year) are also high reaching up to 22% for acute HF.^7^ Owing to the high risk of mortality, modifiable prognostic factors must be identified which can be targeted to reduce the incidence of HF.

Depression is the most common mental disorder that causes significant disability and reduced life satisfaction, especially in the elderly.^8^ The prevalence of depression is variable and depends on the scale used. It is suggested that worldwide prevalence is about 2-19% for minor depression, 1-16% for major depression and 7.2-49% for depressive symptoms.^9^ Depression is frequently noted with other diseases, especially with psychiatric disorders like anxiety & panic disorder, social phobia, eating disorder, etc.^10^ It is also a common condition seen in patients with CVD. Research shows that it is also a risk factor for new-onset CVD and all-cause mortality.^11^ Importantly, a strong link has been found between depression and HF wherein a bi-directional association between the two conditions has been demonstrated.^12^ Numerous mechanisms are thought to play a role in the increased risk of HF in depression patients. Heart rate variability, low-grade systemic inflammation and hypothalamic-pituitary-adrenal axis dysfunction noted in depression patients may increase the risk of HF.^13^ If depression associated with increased development of HF, such patients can be screened early to mitigate the risk and avoid morbidity and mortality of HF. However, the evidence linking the two remains inconclusive.

Previously, Correll et al.^14^ in a meta-analysis of cohort and cross-sectional studies have shown that major depressive disorder is an independent risk factor for CVD, cerebrovascular disease, coronary artery disease and HF. However, their review could include just two cohort studies examining the link between depression and HF. Another review by Cao et al.^15^ has also shown that depression is a risk factor for HF. Nevertheless, just six studies could be pooled in their meta-analysis. Given the small number of studies included in previous reviews and the publication of new data in the literature, there was a need for improved evidence examining the association between these two illnesses. Herein, we present an updated systematic review and meta-analysis of cohort studies assessing if depression is a risk factor for HF.

## METHODOLOGY

We conducted the present review based on the PRISMA guidelines.^16^ All review authors contributed to developing the study protocol which was registered on the database of the International Prospective Register of Systematic Reviews (PROSPERO registration number: CRD42024573423). All reviewers jointly formulated the inclusion criteria before the database search to include only good-quality studies.

### Inclusion criteria:

The current study included only cohort studies conducted on the general population (with or without age restriction) with the exposure variable as depression and the outcome variable as HF. Studies were to exclude those with baseline HF. Studies were to identify depression with objective tests like depression scales or from medical records or by standardized psychiatric interviews. Similarly, HF was to be identified from medical records or by clinical diagnosis. Follow-up was to be ≥5 years and studies were to report multivariable adjusted effect size of the association with 95% confidence intervals (CI).

### Exclusion criteria:

We excluded case-control studies, cross-sectional studies, studies not excluding baseline HF patients, studies with <5 years of follow-up, studies with self-reported depression (without any validated scale) or HF, studies not reporting separate data on HF and studies with duplicate or overlapping data. In such cases, the study with the maximum sample size or with the most covariate adjustments was chosen after discussion between the reviewers. We also did not include unpublished data and studies available only as abstracts.

### Search & Study Selection:

The search strategy was vetted by all authors and was undertaken by two reviewers (LZ & QW) separately. A web-based search was undertaken for PubMed, Embase, CENTRAL, Web of Science and Scopus for all studies published up to 28 July 2024. A separate search of Google Scholar was also undertaken to include grey literature. Keywords used in the search were: cardiac failure, heart failure, depression, depressive symptoms, depressive disorder and cohort. Details of the search strategy are presented in Supplemental Table-I. The reviewers also explored the reference lists of included articles to look for further eligible studies.

**Supplementary Table-I:** Search strategy.

***PubMed, CENTRAL & Web of Science:*** (((cardiac failure) OR (heart failure)) AND (((depression) OR (depressive symptoms)) OR (depressive disorder))) AND (cohort)
***Embase:***
1. ’heart failure’/exp OR ’heart failure’
2. cardiac AND failure
3. #1 OR #2
4. ’depression’
5. ’depressive symptoms’
6. ’depressive disorder’
7. #4 OR #5 OR #6
8. ‘cohort analysis’
9. #3 AND #7 AND #8
***Scopus:***
((TITLE-ABS-KEY (heart AND failure)) OR (TITLE-ABS-KEY (cardiac AND failure ) ) AND ( ( TITLE-ABS-KEY ( depression ) ) OR ( TITLE-ABS-KEY ( depressive AND symptoms ) ) OR ( TITLE-ABS-KEY ( depressive AND disorder ) ) ) AND ( TITLE-ABS-KEY ( cohort ) )

Once the search was completed, articles from all databases were combined and deduplicated by automation. The initial and final screening was undertaken by the two authors (LZ & QW) by examining the titles/abstracts and full texts respectively. Assessment of selected articles regarding inclusion criteria was undertaken independently by two authors (LZ & QW) and subsequently verified by the senior author (HG). All disagreements were discussed and resolved.

### Data extraction and assessment of quality:

Two investigators (LZ & QW) were independently involved in data collection. We extracted information on study authors, study database and location, design, participants, sample size, age, gender details, diabetes mellitus (DM), HT, baseline CVD, current smoking, body mass index (BMI), assessment of depression and HF, prevalence of depression, incidence of HF, follow-up, adjusted covariates and effect size. All definitions of depression were eligible for the present review provided they were reported. If multiple models were used to report the effect size, we chose the one that had the maximally adjusted. If a study reported different subgroups of the cohort, then a composite effect size of the entire cohort was calculated in the meta-analysis software itself for the final analysis. Two reviewers (LZ & QW) used the Newcastle-Ottawa Scale (NOS)^17^ to assess the quality of the included studies. This scale assesses patient selection, comparability between cohorts and outcome analysis as a means of determining study quality, with a maximum score of nine assigned to studies of the highest quality. Score of 7-8 indicate medium and ≤6 indicate low quality.

### Statistical analysis:

The association between depression and HF was calculated by obtaining pooled adjusted hazard ratios (HR) and 95% CI using the DerSimonian-Laird meta-analysis method. It was conducted using the generic inverse variance function of Review Manager (Revman) version 5.3 (Nordic Cochrane Centre, Copenhagen, Denmark). The meta-analysis was conducted using the random-effects model given the anticipated variations in the study populations, disease severity and methodology of the included articles. The unobserved heterogeneity across studies can be accounted by the random-effect model leading to more efficient and generalizable estimates.^18^ Publication bias was examined by visual inspection of the funnel plot.

Heterogeneity was investigated by calculation of the I^2^ statistic, which provides the percentage variability attributed to heterogeneity rather than sampling error. Values of more than 50% indicated significant heterogeneity. To ascertain sources of heterogeneity across studies, we performed subgroup analyses stratified by location, design, included participants, method of assessment of depression and HF, the prevalence of depression, the incidence of HF and follow-up duration. We also conducted a sensitivity analysis to assess the effect of each study on HR. Individual studies were excluded and the HR was recalculated. Meta-regression analysis was conducted using meta-essentials (https://www.erim.eur.nl/research-support/meta-essentials/).

We explored if baseline moderators namely, number of patients with DM, HT, current smoking and mean BMI contribute to differences in study results. These moderators were tested in a random-effects meta-regression against the study results. Outcome was presented as beta coefficient with 95% CI. A positive Beta indicated that higher percentage of patients with DM, HT, current smoking and higher baseline mean BMI was associated with increase in effect size, indicating a stronger association between depression and HF. We also examined the certainty of evidence using the Grading of Recommendations Assessment, Development and Evaluation (GRADE) tool.

## RESULTS

**Fig.1:**
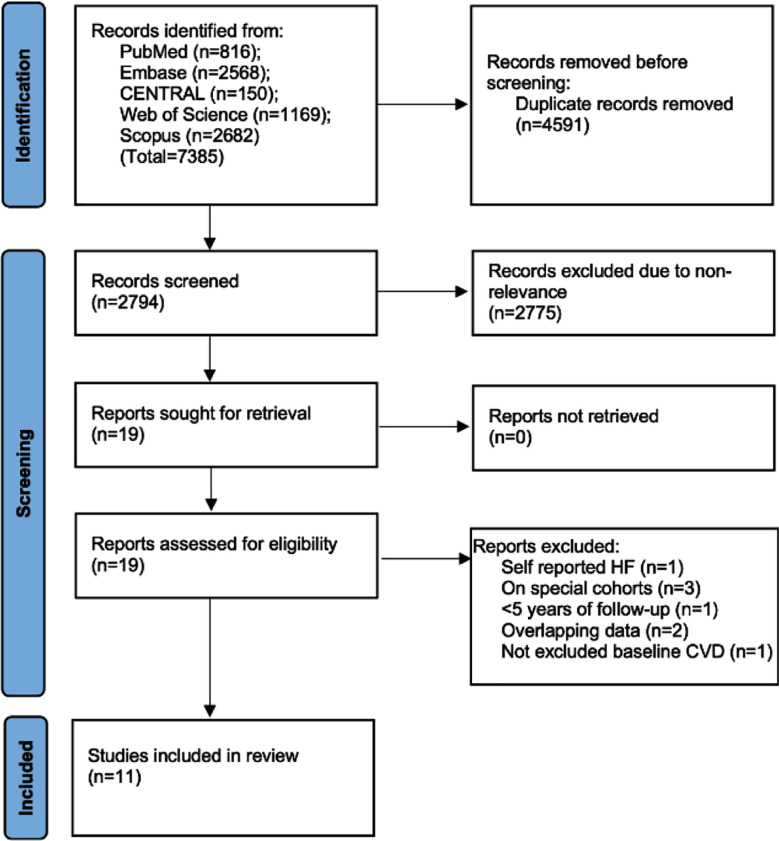
Study flowchart.

The investigators identified 3956 articles after the literature search (Fig.1). Preliminary title/abstract screening was undertaken for 2794 studies of which only 19 made it to the secondary screening process. Eight studies were excluded for different reasons. The remaining 11 studies^19^-^29^ fulfilled the eligibility criteria and were assessed in the present review. Kappa (0.99), denoting inter-reviewer agreement for selection of studies, was high.

### Baseline details:

Data extracted from the included studies can be found in Table-I. Data on the baseline characteristics of participants is shown in Supplementary Table-II. The studies were published between the years 2002 to 2024. Only two were retrospective studies while all others were prospective studies. Most studies originated from the USA while four were from Europe and one was from Asia. Four studies included only elderly adults while others included elderly and middle-aged adults. One study was only on male participants while others included females as well. The combined sample size of the studies was 2,635,205. Diagnosis of depression was based on the Center for Epidemiological Studies Depression Scale, Hospital Anxiety and Depression Scale, geriatric depression scale, medical codes, or prescription of antidepressants. On the other hand, HF was mostly identified from medical records or was physician-diagnosed in two studies. The prevalence of depression varied from 0.7 to 45% while the incidence of HF ranged from 0.03 to 23.6%. Follow-up in the studies varied from 5.8 to 14 years. All studies, except two, were high-quality receiving a score of Eight or Nine on NOS. Two studies received a score of Seven.

**Table-I:** Details of included studies.

Study	Database	Type	Participants, n	Mean age (years)	Males (%)	Assessment of depression and prevalence	Assessment of HF and incidence (%)	Follow-up (years)	Adjusted covariates	NOS score
Williams 2002	Yale Health and Aging Project, USA	P	≥65 years, 2501	74	41.8	CES-D-20 score of ≥21; 7.5	ICD codes, 12.5	14	Age, sex, education, race, marital status, AMI during follow-up, stroke, DM, pulse pressure, hypertension, BMI, smoking, functional status and cognitive function	S-4 C-2 O-2
Kamphuis 2006	Finland, Italy and Netherlands Elderly (FINE) study	P	70-90 years old men, 799	76.5	100	SDS score of ≥50; 32.5	Death certificates, 7.4	NR	Age, country, education, BMI, smoking, alcohol intake, SBP, total and HDL, PA, disability, stroke and DM	S-4 C-2 O-1
van den Broek 2011	Cardiovascular Health Study, USA	P	≥65 years, 4114	72.9	40.8	CES-D-20 score of ≥8; 2	Physician diagnosed, 23.6	10.7	Age, gender, race, SBP, DM, cholesterol level, BMI, smoking status, PA, left ventricular ejection fraction and left ventricular hypertrophy	S-4 C-2 O-1
Gustad 2014	Nord-Trøndelag Health Study, Norway	P	General population, 62567	66.4	46.9	HADS-D score of ≥11; 3.2	ICD codes, 2.4	11.3	Age, sex, marital status, education, smoking, PA, BMI, total cholesterol, DM, resting heart rate, SBP, alcohol, serum creatinine and AMI during follow-up	S-4 C-2 O-3
White 2015	Veterans Aging Cohort Study, USA	P	Veterans, 54519	48.8	97.2	ICD-9 code^1^; 16.1	ICD codes, 3	5.8	Age, sex, race, alcohol abuse, hypertension, DM, smoking, cholesterol, atrial fibrillation, BMI, substance use and antidepressant use	S-4 C-2 O-2
Ogilvie 2016	Multi-Ethnic Study of Atherosclerosis, USA	P	45-84 years, 6782	62.2	NR	CES-D-20 score of ≥16; 12.9	Physician diagnosed, 3.6	9.3	Age, sex, race, field center, smoking, PA	S-4 C-2 O-2
Daskalopoulou 2016	CALIBER programme, UK	R	>30 years, 1937360	NR	49	Depression diagnosis or prescription of antidepressants by Read codes; 18.9	Read codes, 1.2	6.9	Age, gender, smoking, systolic blood pressure, DM, cholesterol and socio-economic status	S-4 C-2 O-3
Dixon 2022	Southern Community Cohort Study, USA	P	40-79 years, 23937	53*	36	CES-D-10 score of ≥10; 45	ICD codes, 45	11	Age, sex, race, hypertension, HLD, DM, BMI, stroke/TIA, smoking, income, education, employment, alcohol, physical activity, marital status, close friends, help in emergency, antidepressants	S-4 C-2 O-3
Khodneva 2022	REGARDS study, USA	P	≥45 years, 21888	63	42.2	CES-D-4 score of ≥4; 9.9	Medical records for HF hospitalization. 9.9	9.2	Age, race, sex, region, education, income, marital status, SBP, BMI, antihypertensives, DM, urinary albumin to creatinine ratio, C-reactive protein, smoking, alcohol use, PA, health insurance, primary care provider, self-reported physical health component score of short form-12 scale and interim nonfatal AMI on/before incident HF	S-4 C-2 O-3
Nakada 2023	UK biobank	P	40-69 years, 431973	54	38.2	ICD codes (F32-33); 0.7	ICD codes, 0.7	Up to 11	Age, sex, ethnicity, deprivation level, alcohol intake, smoking status, sleep duration, television viewing, PA and BMI	S-4 C-2 O-3
Kang 2024	National Health Insurance Service database, Korea	R	66 years, 88765	66	51.5	Questions from GDS^2^; 18.2	ICD codes, 18.2	6.8	Sex, age, insurance premium, BMI, smoking, drinking, exercise frequency, SBP, anti-hypertensives, fasting glucose level, diabetes medication, total cholesterol level, lipid-lowering medication, antidepressants and Charlson comorbidity index	S-4 C-2 O-2

AMI, acute myocardial infarction; BMI, body mass index; CVD, cardiovascular disorder; CES-D, Center for Epidemiological Studies Depression Scale; DM, diabetes mellitus; HADS-D, Hospital Anxiety and Depression Scale; GDS, geriatric depression scale; HDL, high-density lipoprotein; HF, heart failure; ICD, International Classification of Diseases; LDL, low-density lipoprotein; PA, physical activity; SBP, systolic blood pressure; SDS, Self-rating Depression Scale; NR, not reported; NOS, Newcastle Ottawa scale; TIA, transient ischemic attack; P, prospective; R, retrospective; S-selection of cohort; C, comparability of groups; O, outcome assessment,

1 Considered to have depression if at least 1 inpatient or 2 outpatient ICD-9 codes for depression (296.2x and 296.3x) were identified

2 To evaluate depressive symptoms of participants, three yes/no questions selected from the Geriatric Depression Scale. Each of the three yes/no questions measured loss of activities and interests, worthlessness and hopelessness. The detailed questions were as follow: “Have you dropped many of your activities and interests?”, “Do you feel pretty worthless the way you are now?” and “Do you feel that your situation is hopeless?”. Participants who answered affirmatively to any of these yes/no questions were classified as having depressive symptoms.

*Median value

**Supplementary Table-II:** Baseline medical characteristics of the included samples.

Study	DM (%)	HT (%)	Current smoking (%)	CVD (%)	Mean BMI
Williams 2002	12.2	41	19.8	9.2	NR
Kamphuis 2006	NR	NR	27	NR	NR
van den Broek 2011	17.7	NR	10.9	17.7	26.6
Gustad 2014	99.8	NR	29.3	3	NR
White 2015	20.5	41.3	54	NR	NR
Daskalopoulou 2016	3.9	NR	11.8	NR	27
Ogilvie 2016	12.6	37.3	13	NR	29
Dixon 2022	25.3	61	40.8	NR	NR
Khodneva 2022	24.9	70.9	NR	0	29
Nakada 2023	NR	NR	10	NR	28
Kang 2024	15.5	46.9	11.8	NR	24.2

DM, Diabetes mellitus; HT, hypertension; CVD, cardiovascular disease; BMI, body mass index; NR, not reported

### Analysis:

**Fig.2:**
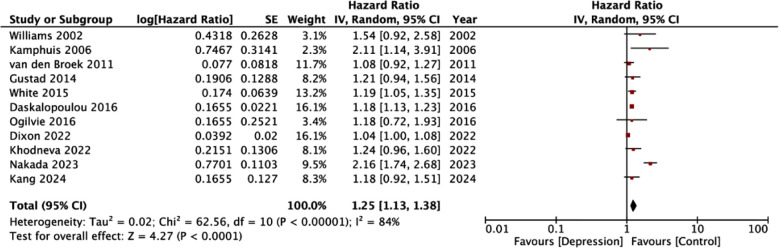
Meta-analysis for the association between depression and HF.

**Fig.3:**
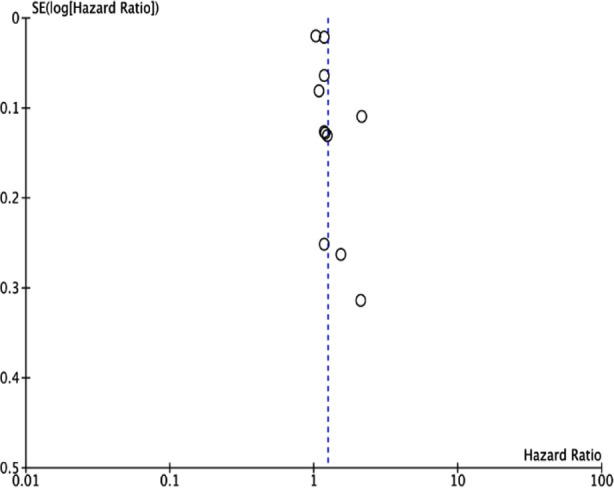
Funnel plot for publication bias.

A pooled analysis of all included studies denoted that depression was a statistically significant risk factor for the development of HF (HR: 1.25 95% CI: 1.13, 1.38) (Fig.2). We could note high inter-study heterogeneity in the meta-analysis (I^2^=87%). Visual inspection of the funnel plot did not demonstrate any publication bias (Fig.3).

Sensitivity analysis was conducted by eliminating one study at a time to examine if the results changed in significance on the exclusion of any singular cohort. Results are presented in Table-II. All resultant HRs were statistically significant and ranged from 1.15 to 1.30. The heterogeneity did not reduce significantly on the exclusion of any study.

**Table-II:** Sensitivity analysis.

Study	Resultant HR (95% CI)	I^2^
Williams 2002	1.24 [1.12, 1.37]	85
Kamphuis 2006	1.23 [1.11, 1.36]	85
van den Broek 2011	1.27 [1.14, 1.42]	86
Gustad 2014	1.25 [1.12, 1.39]	86
White 2015	1.26 [1.13, 1.41]	85
Daskalopoulou 2016	1.29 [1.11, 1.49]	83
Ogilvie 2016	1.25 [1.13, 1.39]	86
Dixon 2022	1.30 [1.15, 1.47]	74
Khodneva 2022	1.25 [1.12, 1.39]	85
Nakada 2023	1.15 [1.07, 1.25]	66
Kang 2024	1.26 [1.13, 1.40]	86

HR, hazard ratio; CI, confidence intervals.

The results of the subgroup analysis can be found in Table-III. Based on location, we noted that the association between depression and HF was significant for studies from the USA and Europe. The lone Asian study found non-significant results. Based on the study design, results were significant for both prospective and retrospective studies. The HR was also statistically significant for studies including only the elderly and those including a mixed population. The method of assessment of depression did not have a major effect on the results while subgroup analysis of studies using physician-diagnosed HF showed non-significant results. There was no change in the significance of results based on prevalence of depression (>10% or <10%) but subgroup analysis of studies with >10% incidence of HF showed marginally non-significant results. The HRs were significant in both studies with follow-ups of >10 years and <10 years.

Meta-regression results are shown in Table-IV. The beta coefficients for the meta-regression were 0.0002, -0.0035, -0.0064 and 0.0692 for the moderators baseline DM, HT, current smoking, mean BMI respectively. All of the 95% CI were statistically non-significant. GRADE assessment of evidence is presented in Supplementary Table-III. The certainty of evidence was found to be “very low”.

**Table-III:** Subgroup analysis.

Variable	Subgroup	Studies	Hazard ratio [95% confidence intervals]	I^2^
Location	USA	6	1.11 [1.02, 1.20]	37
	Europe	4	1.53 [1.09, 2.16]	
	Asia	1	1.18 [0.92, 1.51]	91 -
Design	Prospective	9	1.30 [1.11, 1.53]	85
	Retrospective	2	1.18 [1.13, 1.23]	0
Participants	Only elderly	4	1.25 [1.01, 1.55]	46
	Mixed	7	1.25 [1.11, 1.41]	89
Assessment of depression	Clinical scales	7	1.14 [1.02, 1.28]	42
	Medical codes	3	1.41 [1.09, 1.83]	93
	Others	1	1.18 [0.92, 1.51]	-
Assessment of heart failure	Medical records	9	1.28 [1.14, 1.43]	87
	Physician diagnosed	2	1.09 [0.94, 1.27]	0
Prevalence of depression	>10%	6	1.15 [1.05, 1.26]	79
	<10%	5	1.39 [1.04, 1.85]	85
Incidence of heart failure	>10%	3	1.05 [0.98, 1.13]	17
	<10%	7	1.36 [1.15, 1.60]	81
Follow-up	>10 years	5	1.32 [1.02, 1.72]	91
	<10 years	6	1.19 [1.14, 1.23]	0

**Table-IV:** Meta-regression analysis.

Moderator	Beta	SE	-95% CI	+95% CI	p-value
Diabetes Mellitus	0.0002	0.0018	-0.0039	0.0043	0.91
Hypertension	-0.0035	0.0097	-0.0284	0.0214	0.71
Current smoking	-0.0064	0.0057	-0.0194	0.0066	0.26
Mean BMI	0.0692	0.0664	-0.1016	0.2400	0.29

BMI, Body mass index; CI, confidence intervals; SE, standard error

**Supplementary Table-III:** GRADE assessment of evidence.

Certainty assessment							Summary of findings				
Participants (studies) Follow-up	Risk of bias	Inconsistency	Indirectness	Imprecision	Publication bias	Overall certainty of evidence	Study event rates (%)		Relative effect (95% CI)	Anticipated absolute effects	
							With control	With Depression		Risk with control	Risk difference with Depression
11 cohort studies	serious^a^	not serious	not serious	not serious	none	⨁◯◯◯ Very low^a^	11 participants	11 participants	HR 1.25 (1.13 to 1.38) [New Outcome]	Low	
										0 per 1,000	-- per 1,000 (from -- to --)

CI: confidence interval; HR: hazard ration, Explanations: a. Only five of the 11 studies received a score of 9 on Newcastle Ottawa Scale. Remaining got a score of 7 or 8.

## DISCUSSION

In recent years, there has been increased awareness of the impact of psychological health on CVD. Particularly, the association between depression and the risk of CVD has been a subject of research in the past decade. Depression has been linked with the development of CVD as well as worse outcomes in those with pre-existing disease.^11^,^30^ A large study by Harshfield et al.^30^ with pooled individual patient data analysis of 563,255 participants from 22 cohorts has found that individuals with baseline depressive symptoms are at an increased risk of CVD. The risk is persistent even at symptom levels lower than the threshold indicative of a depressive disorder. Ghafor et al.^11^ in a recent meta-analysis of 26 studies have shown that depression was associated with an increased risk of stroke, myocardial infarction, HF and CVD. Despite such large studies, questions have been raised on the validity of results given a large number of low-quality studies in the literature.^31^ Given the soaring burden of cardiac diseases globally, there is a need for high-quality evidence to establish a clear link between depression and specific cardiac ailments so that targeted interventions can be developed to mitigate such risks. In the current study, we aimed to assess the link between depression and HF by formulating inclusion criteria that would allow only good to high-quality studies for a meta-analysis.

Our meta-analysis of about 2.6 million participants showed that patients with depression had a 25% increased risk of HF. This risk ranged from about 13% to as high as 38%. Supporting the results of our review was the outcome of sensitivity analysis which found no outlier study. Sequential exclusion of individual studies still supported an increased risk of HF ranging from 15 to 30%. Our results are in agreement with the previous review of Cao et al.^15^ who also noted an increased risk of HF in depression patients with an HR of 1.23 (95% CI: 1.08, 1.41). However, their review could include just six studies with about 1.3 million participants. By including five more studies and doubling the number of participants compared to the prior review, we believe our study significantly increases the statistical power of the meta-analysis thereby providing the best possible evidence in the literature.

Our results also draw parallels with evidence linking depression with other CVDs. Wu et al.^32^ in a meta-analysis of prospective cohort studies have shown a 31% increased risk of myocardial infarction and a 36% increased risk of coronary death in depressed individuals. Lin et al.^33^ in their meta-analysis of 15 studies have shown an increased risk of coronary artery calcification with depression. Furthermore, depression has also been found to increase the risk of adverse events in patients undergoing percutaneous coronary intervention and coronary artery bypass surgery.^34^,^35^

High inter-study heterogeneity was seen in the meta-analysis with I^2^=87%. We attempted to explore the source of this heterogeneity by conducting several subgroup analyses. It was noted that study location, study design, age of participants, method of assessment of depression, prevalence of depression and follow-up did not have a major effect on the significance of the results. We noted a few non-significant results for studies with physician-diagnosed HF and those with >10% incidence of HF. However, such subgroups had very few studies and it been plausible that the statistical power of these subgroups was not high enough to demonstrate any significant results. Despite the multiple subgroup analyses, we were unable to deduce the source of inter-study heterogeneity. It is possible that variations in the study populations, comorbidities, severity of depression and other unknown confounders could have contributed to the high heterogeneity in the meta-analysis. Notably, although adjusted data from the studies were utilized, the covariates employed by each study differed based on the baseline data accessible for the cohorts.

We still selected models with the most adjusted data to account for maximum covariates and reduce association confounding. We believe this provides more robust outcomes rather than using models that exclude known confounders, leading to a biased association between exposure and outcomes. Furthermore, due to the studies being conducted in various locations with differing data collection methods, the adjusted covariates could not be uniform across the studies.

An important confounder for our results is the method of identification of depression. While we noted significant results for studies using clinical scales and medical codes, the type of scales used for depression varied amongst studies. More so the cut-off for the diagnosis of depression was also variable. Such variations can exclude patients with mild to moderate depression and therefore alter the results of the association. Kamphuis et al.^25^ have found a dose-response relationship between depressive symptoms and CVD indicating that patients with severe depression are at a higher risk of HF. Likewise, Gustad et al.^21^ have found that patients with the Hospital Anxiety and Depression Scale score of ≥11 had a higher risk of HF as compared to those with a score of 8-10. Due to the limited data and variation in depression scales, we were unable to establish such a dose-response relationship in our meta-analysis. However, it needs to be investigated by further studies. Another important factor for consideration was the effect of baseline patient characteristics on the risk of HF. While our meta-regression analysis failed to demonstrate a statistically significant association between baseline DM, HT, current smoking, mean BMI and the pooled effect size, data was limited and hence results must be interpreted with caution.

Several potential mechanisms can explain the increased risk of HF in depressed patients. Research shows that impaired heart rate variability seen in depression can accelerate coronary atherosclerosis thereby inducing myocardial ischemia, ventricular dysrhythmias and subsequently HF.^13^,^36^ Depression is also independently linked with low-grade systemic inflammation which in turn contributes to CVD.^37^ Depressed patients also have elevated cortisol levels which demonstrates hypothalamic-pituitary-adrenal axis dysfunction due to psychological stress. Hypothalamic-pituitary-adrenal axis dysfunction is a potential contributor to CVD.^38^ Another mechanism is endothelial dysfunction which is noted in depression even without in the absence of cardiac risk factors. Healthy endothelium is critical for cardiovascular health and dysfunction is seen even before the first incidence of CVD.^39^

Furthermore, several behavioral changes seen with depression can also increase the risk of HF. Depressed patients are more prone to adverse habits like smoking, alcohol intake and physical inactivity and are less likely to adhere to medications, healthy diet and rehabilitation programs which can compromise the individual’s health status and hence contribute to HF.^15^ There is also a possibility of an indirect effect of depression on HF via other comorbidities. Depression is a known risk factor for DM,^40^ HT^41^ and coronary artery disease^32^ which in turn are risk factors for HF.

### Strength of the Review:

The strengths of our review include the inclusion of only cohort studies with a large sample size of 2.6 million participants. We excluded cross-sectional and case-control studies as only cohort studies can establish a temporal relationship between depression and HF thereby providing high-quality evidence. We also formulated the inclusion criteria to exclude studies that did not eliminate baseline HF patients from the cohort, thereby minimizing reverse causality. Studies with self-reported depression without the use of validated scales and self-reported HF were also not included to reduce the risk of recall bias. Furthermore, only studies with sufficiently long follow-up (>5 years) were analyzed to provide better evidence.

### Limitations:

Methodological variations in the assessment of depression and HF among studies are a major drawback. As mentioned earlier, the prevalence of depression can vary significantly with the type and cut-off of the scale.^9^ Secondly, while we used only adjusted HRs, unmeasured confounding is a possibility as the adjusted covariates were not similar in the included studies. Thirdly, we were unable to stratify the risk of HF based on the severity of depression due to a lack of adequate data. Fourthly, most of the data was from the Western population and there is limited data on Asian and African populations. Cultural, socioeconomic, and healthcare access factors could significantly influence both depression diagnosis and HF risk. Hence, the results cannot be generalized for the global population. We were also unable to examine the effect of population characteristics like gender, ethnicity, cultural background and social determinants of health on the outcomes. Hence, it is unclear if our results could be generalized to all populations. Moreover, data segregated based on ejection fraction was not available from most studies. Only Khodneva et al.^20^ divided their cohort based on ejection fraction and reported that depression was associated with increased risk of developing HF with preserved ejection fraction but not with HF with reduced or midrange ejection fraction. Further studies are needed to clarify such relationship. Fifthly, there was little information on the treatment of depression in the included studies. While some studies adjusted for antidepressant use, it was unclear if treatment of depression mitigated the risk of HF. Moreover, drugs like tricyclic antidepressants have proven cardiotoxic effects^42^ and it is plausible that use of such drugs could have led to increased risk of HF. Lastly, we did not use specific search terms like cardiomyopathy, HF with reduced or preserved ejection fraction and major depressive disorder during the literature search. Instead, broader search terms were used to allow for maximum number of results so that the reviewers could manually screen for relevant articles.

### Clinical implications:

The findings of our review hold significant ramifications for public health. Depression must be regarded as a significant risk factor for HF in clinical practice. Upon establishing a diagnosis of depression, patients and caregivers must be apprised of the increased risk of HF. Other HF risk factors like coronary artery disease, HT, DM and obesity should be promptly recognized in these patients with should be treated appropriately to avoid an extended risk of HF. Healthcare professionals must advocate healthy lifestyle practices, including frequent physical activity, a nutritious diet and stress reduction strategies, to enhance both mental and cardiovascular well-being. These patients may also benefit from prompt referral to a cardiologist, who can regularly monitor for indications of heart failure, allowing for timely intervention to mitigate morbidity and mortality associated with the condition.

To overcome the limitations of the review, we recommend further studies using standardized depression scales and segregating patients based on the severity of depression to further improve evidence. Studies should also report data from Asian and African populations for better generalization of outcomes.

## CONCLUSIONS

The present results show that there seems to be an association between depression and development of HF. However, the certainty of evidence in very low.

### Authors’ contributions:

**HG:** Literature search, study design and manuscript writing.

**LZ and QW:** Data collection, data analysis and interpretation. Critical Review.

**HG:** Critical analysis, Manuscript revision and validation and is responsible for the integrity of the study. All authors have read and approved the final manuscript.
